# In situ Degradation and Characterization of Endosperm Starch in Waxy Rice with the Inhibition of Starch Branching Enzymes during Seedling Growth

**DOI:** 10.3390/ijms19113397

**Published:** 2018-10-30

**Authors:** Ting Pan, Lingshang Lin, Qiaoquan Liu, Cunxu Wei

**Affiliations:** 1 Key Laboratory of Crop Genetics and Physiology of Jiangsu Province/Key Laboratory of Plant Functional Genomics of the Ministry of Education, Yangzhou University, Yangzhou 225009, China; m150776@yzu.edu.cn (T.P.); 18252713442@163.com (L.L.); qqliu@yzu.edu.cn (Q.L.); 2 Co-Innovation Center for Modern Production Technology of Grain Crops of Jiangsu Province/Joint International Research Laboratory of Agriculture & Agri-Product Safety of the Ministry of Education, Yangzhou University, Yangzhou 225009, China

**Keywords:** waxy rice, starch branching enzyme, seedling growth, starch, in situ degradation, structural properties

## Abstract

High-resistant starch cereal crops with the inhibition of the starch branching enzyme (SBE) have been widely studied. However, the effects of the inhibition of SBE on waxy cereal crops are unclear. A transgenic rice line (GTR) derived from a *japonica* waxy rice cultivar Guang-ling-xiang-nuo (GLXN) has been developed through antisense RNA inhibition of both SBEI and SBEIIb. In this study, GLXN and GTR were cultivated in the dark only in deionized H_2_O, and their shoot and root growth, starch in situ degradation, and starch property changes were investigated during seedling growth. Compared with GLXN, GTR showed a significantly slow seedling growth, which was not due to the embryo size and vitality. The slow degradation of starch in the seed restrained the seedling growth. GLXN starch was completely degraded gradually from the proximal to distal region of the embryo and from the outer to inner region in the endosperm, but GTR starch in the peripheral region of the endosperm was not completely degraded, and the starch residual was located in the outside of the compound starch though its degradation pattern was similar to GLXN. During seedling growth, GLXN starch had the same A-type crystallinity and a similar ordered structure, but the crystallinity changed from the C_A_-type to B-type and the ordered structure gradually increased in the GTR starch. The above results indicated that GTR had a heterogeneous starch distributed regionally in the endosperm. The starch in the peripheral region of the endosperm had a B-type crystallinity, which was located in the outside of the compound starch and significantly increased the resistance to in situ degradation, leading to the seedling slow growth.

## 1. Introduction

Starch is the major storage carbohydrate in cereal endosperm and provides energy and nourishment for humans. In normal cereal crops, starch mainly consists of amylose and amylopectin. The content and structure of amylose and amylopectin determine the physicochemical properties of starch and influence the applications of cereal seeds [1,2]. Amylose in cereal endosperm is mainly synthesized by granule-bound starch synthase I (GBSSI), and amylopectin is mainly synthesized by the soluble starch synthase, starch branching enzyme (SBE), and starch debranching enzyme [3]. The loss-of-function mutant of GBSSI can produce waxy crops composed of amylose-free starch and influence the eating and cooking quality of cereal seeds [3]. Suppressing or eliminating SBE activities in cereal endosperm can change the amylopectin structure, decrease amylopectin synthesis, and increase the amylose content, leading to a significant increase in resistance starch (RS) [4,5,6,7,8,9,10]. RS is an important starch component, which cannot be digested in the upper gastrointestinal tract but functions as a substrate for bacterial fermentation in the large intestine [11,12]. Foods with high RS have health benefits including lowering glycemic and insulin responses and reducing the risk of developing type II diabetes, obesity, and cardiovascular disease [11,12]. Therefore, many high-RS crops have been cultivated via inhibition of SBE expression or mutation of SBE genes [4,5,6,7,8,9,10].

For cereal crops, endosperm starch provides energy and nutrition for grain germination and seedling growth. For normal cereal crops, seed starch can be homogeneously degraded gradually from the embryo and aleurone layer during seedling growth [10,13,14]. However, for the maize *sbe I* mutant with changed amylopectin structure and amylose content, seed starch is resistant to amylase hydrolysis during grain germination and seedling growth, leading to the inhibition of seedling growth [13]. A high-amylose and RS rice line with the expression inhibition of SBEI/IIb has a 60% amylose content and a 14.6% RS in endosperm [9,15]. Its amylopectin branching degree decreases and the branch-chain lengthens, leading to a crystalline structure change from A-type to C-type [15]. During the seedling growth in the dark only in deionized H_2_O, the slow degradation of seed starch, which is due to the high resistance of amylopectin long branch-chains and B-type crystallinity to in situ degradation, restrains the growth of the shoot and root [14]. Shaik et al. [10] thought that when the starch granule and molecular structure are changed, its amylase hydrolysis properties also change during grain germination and seedling growth and further influence the remobilization of seed storage material and the establishment of the seedling.

So far, the high-RS cereal crops with the inhibition of SBE expression or mutation of SBE genes all have high amylose content [16]. The high amylose in endosperms significantly influences the eating and cooking quality, and inhibits the in vivo digestion of starch when fed as foods and the in situ degradation during seedling growth [4,5,6,7,8,9,10,14]. However, the effects of the inhibition of SBE expression or mutation of SBE genes on waxy cereal crops are unclear.

A transgenic rice line (GTR) derived from a *japonica* waxy rice cultivar Guang-ling-xiang-nuo (GLXN) has been developed through antisense RNA inhibition of both SBEI and SBEIIb [9]. The deficiency of GBSSI leads to no amylose synthesis in both GLXN and GTR [17]. In GTR, the expression and activity of SBEI, SBEIIa, and SBEIIb were significantly declined according to the results of the reverse transcription-PCR analysis, immunoblotting assay, and native PAGE/activity staining of starch biosynthesis-related enzymes in developing endosperm [18]. The reduced expression of SBEIIa might be the antisense inhibition designed for *SBEIIb* to also play a role in *SBEIIa* expression due to the sequence similarity [18,19]. In this study, the shoot and root growth, starch in situ degradation, and starch property changes of GLXN and GTR were investigated during seedling growth in the dark and only in deionized H_2_O. Our objective was to reveal the in situ degradation and characterization of endosperm starch in waxy rice with inhibition of SBE during seedling growth.

## 2. Results and Discussion

### 2.1. Growth Dynamics of Rice Seedlings in the Dark Only in Deionized Water

**Figure 1:**
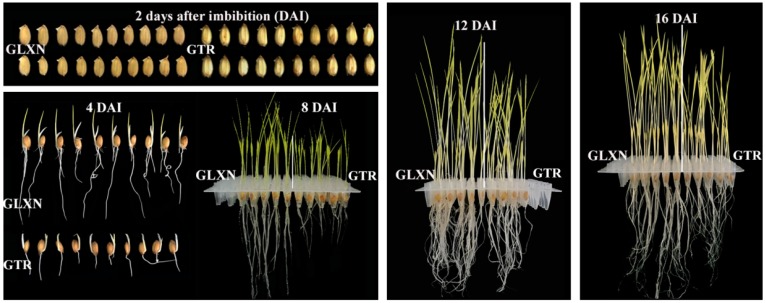
Photographs of rice grain germination and seedling growth in the dark only in deionized H_2_O.

**Figure 2:**
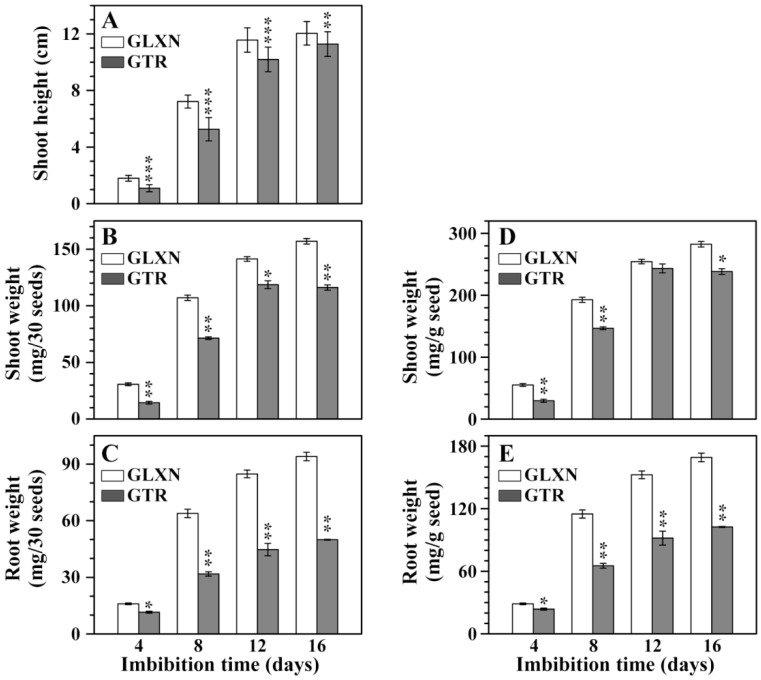
The shoot height (**A**), shoot dry weight (**B**,**D**), and root dry weight (**C**,**E**) of rice seedling. The values are means ± SD (*n* = 3). * The GTR data are significantly different compared with the GLXN data at the same imbibition time (* for *p* < 0.05, ** for *p* < 0.01, and *** for *p* < 0.001).

In order to ensure that the energy and nutrient substance needed for grain germination and seedling growth were provided only through degradation of seed materials, rice grains were cultivated in the dark only in deionized H_2_O according to the method by Shaik et al. [10]. It was noteworthy that the deficiency of mineral elements such as calcium and potassium could influence seedling growth, but this growing condition had effects on the in situ degradation and characterization of endosperm starch during seedling growth. Figure 1 shows the grain germination and seedling growth in the dark only in deionized H_2_O. The grain germination began from 2 days after imbibition (DAI) and was slightly slower in GTR than in GLXN. The seedling growth, including shoot height and root length, was significantly slower in GTR than in GLXN from 4 DAI. The shoot height, shoot weight, and root weight were further quantitatively measured on the 30 seed basis and on the same weight basis of pre-germinated dry seeds (Figure 2). The shoot height, shoot weight, and root weight was significantly lower in GTR than in GLXN, indicating that the GTR seed materials were more resistant to degradation and provided less energy and nutrient substance for seedling growth than did the GLXN seed materials. The present results agreed with the previous reports that the repression of SBE expression in endosperm can inhibit seedling growth [10,14].

### 2.2. In Vitro Culture of Mature Embryo

**Figure 3:**
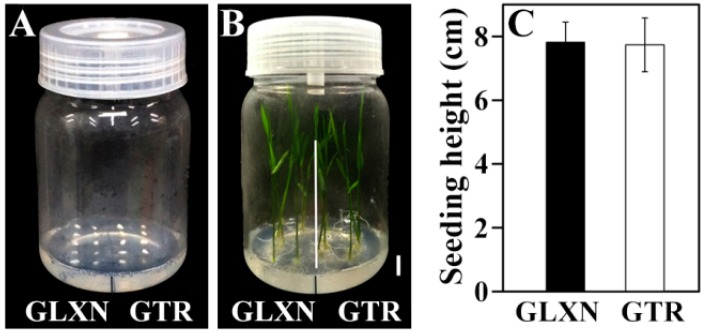
The in vitro culture of the mature embryo. (**A**,**B**): the 0 and 6 days after in vitro culture, respectively, scale bar = 1 cm; (**C**): the shoot height at 6 days after in vitro culture. The values are means ± SD (*n* = 30), and the GTR data have no significant difference from the GLXN data (*p* = 0.785).

In order to exclude whether the slow growth of GTR seedling resulted from the embryo size and vitality, the in vitro culture of the mature embryo was performed (Figure 3). No significant difference was detected in seedling growth at 6 days after in vitro culture between GLXN and GTR. This result demonstrated that the slow growth of the GTR seedling resulted from the slow degradation of seed materials and was not due to the embryo size and vitality. A similar result was also reported by Pan et al. [14].

### 2.3. The Consumption of Seed Material and Its Relationship with Seedling Growth

**Figure 4:**
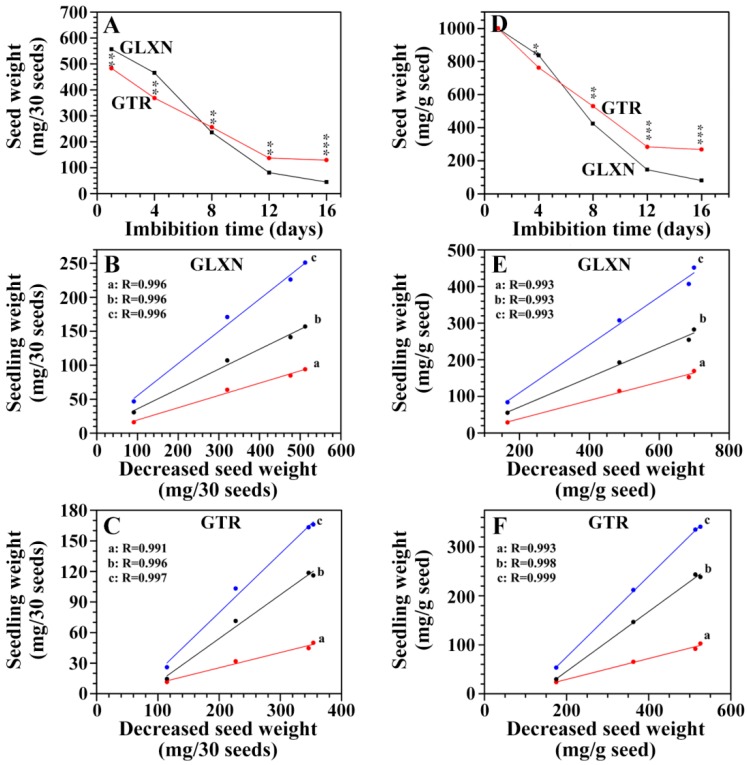
The seed weight and its relationship with seedling weight during seedling growth. (**A**,**D**): dry weight of seed without the embryo. The values are means ± SD (*n* = 3). * The GTR data are significantly different compared with the GLXN data at the same imbibition time (** for *p* < 0.01 and *** for *p* < 0.001). (**B**,**C**,**E**,**F**): the relationships between the decreased seed weight and root dry weight (a), shoot dry weight (b), and seedling dry weight (root weight + shoot weight) (c). The R indicates the regression coefficient.

The seed dry weight was measured during seedling growth on a 30 seed basis and on the same weight basis of pre-germinated dry seed (Figure 4A,D). The decrease of seed weight was significantly slower in GTR than in GLXN, leading to the seed weight being higher in GTR than in GLXN after 12 DAI though the seed weight was higher in GLXN than in GTR before germination. The seed biomass is remobilized to the emerging root and shoot of seedling [10]. Therefore, the relationships between the decreased seed weight and the root weight, shoot weight, and seedling weight (root and seedling weight) were analyzed (Figure 4B,C,E,F). The high correlation coefficient (*R* ≥ 0.991) showed that the decreased seed weight and seedling growth had a highly positive relationship, and the degraded seed materials were used for seedling growth. Compared with GLXN, the slow growth of the GTR seedling in the dark only in deionized H_2_O resulted from the slow degradation of seed materials. Similar phenomena are also observed in high-amylose rice and barley with an inhibition of SBE expression [10,14].

**Figure 5:**
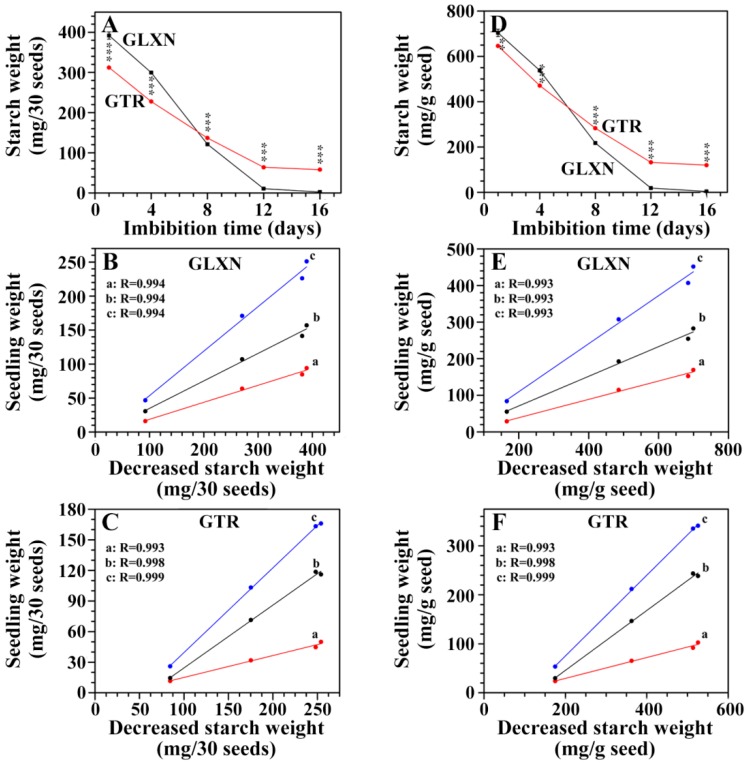
The starch weight in seeds and its relationship with seedling weight during seedling growth. (**A**,**D**): dry weight of starch in seeds. The values are means ± SD (*n* = 3). * The GTR data are significantly different compared with the GLXN data at the same imbibition time (** for *p* < 0.01 and *** for *p* < 0.001). (**B**,**C**,**E**,**F**): the relationships between the decreased starch weight and root dry weight (a), shoot dry weight (b), and seedling dry weight (root weight + shoot weight) (c). The R indicates the regression coefficient.

Starch is the major component in rice seed. The starch weight in the seeds was also measured on a 30 seed basis and on the same weight basis of pre-germinated dry seeds during seedling growth (Figure 5A,D). The starch was rapidly degraded before 12 DAI, but the degradation rate was faster in GLXN than in GTR, indicating that the starch in the GTR seed was more resistant to in situ degradation than the GLXN starch. The high correlation coefficient (*R* ≥ 0.991) also indicated that the degraded starch was used for seedling growth (Figure 5B,C,E,F). It was noteworthy that the endosperm starch in GLXN was completely degraded at 16 DAI, but the endosperm starch in GTR had a similar content between 12 and 16 DAI and maintained a relatively high level. These results showed that residual starch in GTR seeds of 12 and 16 DAI had a high resistance to in situ degradation during seedling growth.

### 2.4. In Situ Degradation of Endosperm Starch during Seedling Growth

**Figure 6:**
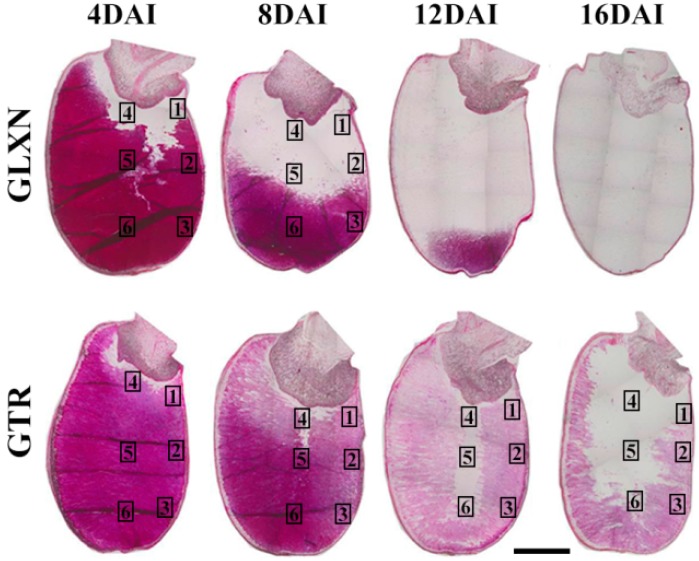
The longitudinal section of the whole kernel during seedling growth. The section is stained with Schiff′s reagent. Scale bar = 2 mm.

**Figure 7:**
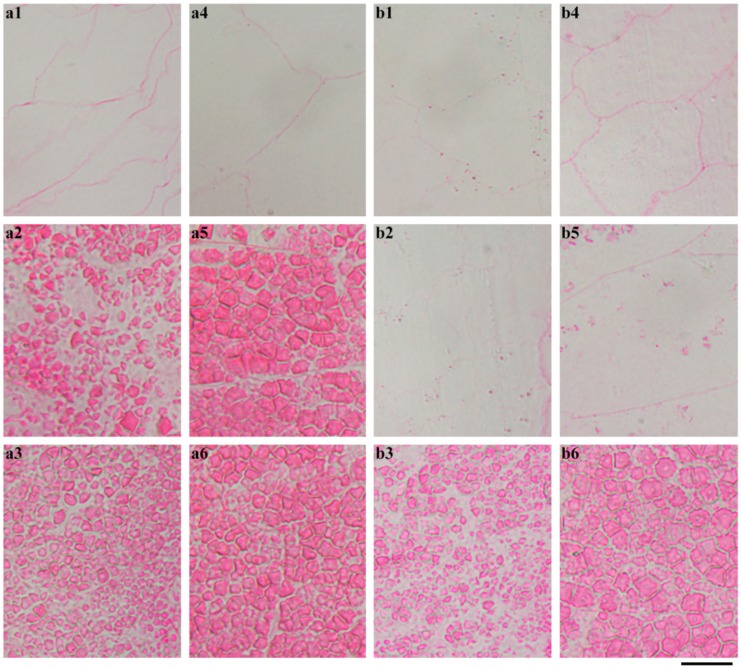
The morphology of endosperm starch in GLXN seed during seedling growth. (**a**) 4 DAI; (**b**) 8 DAI; (1, 2, 3, 4, 5, 6) the magnification of the square area shown in Figure 6. The section is stained with Schiff′s reagent. Scale bar = 20 µm.

The whole seeds were longitudinally sectioned and stained with Schiff′s reagent to exhibit the in situ degradation of starch during seedling growth (Figure 6). The starch was stained red. For GLXN, the whole seed showed that starch was degraded gradually from the proximal to the distal region of the embryo and from the outer to the inner part of the endosperm. At 12 DAI, only a little starch existed in the distal region of the embryo. At 16 DAI, the starch was completely degraded (Figure 6). The region magnification showed that the starch close to embryo had been completely degraded, and that the inner region of the seed and the distal region from that embryo had an intact compound starch at 4 DAI (Figure 7a). The inner region of the seed had been completely degraded and the starch close to the aleurone layer at the distal region from the embryo was partly degraded but the distal region remained intact at 8 DAI (Figure 7b). The above-degraded pattern of endosperm starch in GLXN agreed with that of other normal cereal crops and was due to the amylase which is synthesized in the embryo and the aleurone layer and secreted into the endosperm cells from the outer to the inner layer to degrade starch [10,14]. The present results also indicated that compound starch in different regions of seed could be completely degraded, though their degradation time was different.

**Figure 8:**
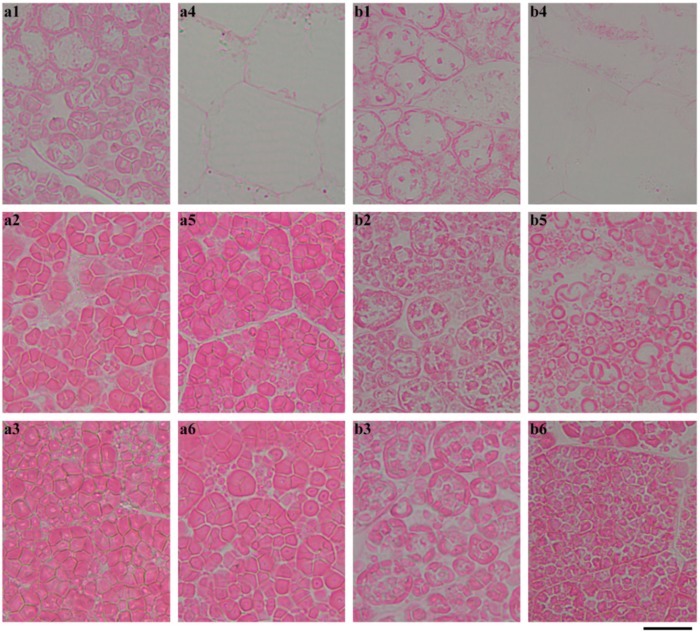
The morphology of the endosperm starch in the GTR seed during seedling growth. (**a**) 4 DAI; (**b**) 8 DAI; (1, 2, 3, 4, 5, 6) the magnification of the square area shown in Figure 6. The section is stained with Schiff′s reagent. Scale bar = 20 µm.

**Figure 9:**
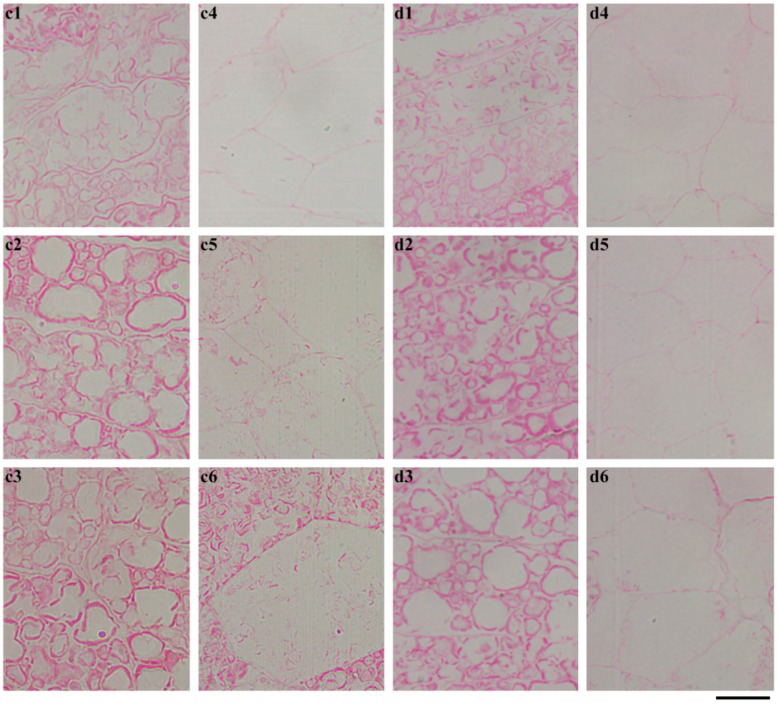
The morphology of the endosperm starch in the GTR seed during seedling growth. (**c**) 12 DAI; (**d**) 16 DAI; (1, 2, 3, 4, 5, 6) the magnification of the square area shown in Figure 6. The section is stained with Schiff′s reagent. Scale bar = 20 µm.

For GTR, the degradation pattern of the starch in seeds was completely different from that of GLXN. The starch was degraded from the periphery of the embryo and proceeded to the distal region of the embryo, which was similar to that of GLXN, but the starch was not completely degraded (Figure 6). The region magnification of the seed showed that the inner endosperm starch close to the embryo was completely degraded but the peripheral endosperm starch close to the embryo was partly degraded at 4 DAI (Figure 8a). At 8 DAI, the endosperm starch in the middle region of the seed began to degrade, but the starch residue in the peripheral region close to the embryo still existed (Figure 8b). At 12 DAI, the inner endosperm starch of seed was completely degraded and that in the distal region from the embryo was also partly degraded. However, the inner parts of the compound starch in the peripheral region of the endosperm were completely degraded, but its outer part was not degraded completely (Figure 9c). At 16 DAI, the inner endosperm starch from the embryo to the distal region was all completely degraded, but the peripheral endosperm starch was not completely degraded (Figure 9d). The present results indicated that the starch in GTR seed was not homogeneously distributed. The high-amylose rice derived from the *indica* rice cultivar Te-qing with an inhibition of the SBEI/IIb expression has polygonal, aggregate, elongated, and hollow starch granules in the endosperm. They were specifically distributed in different regions from the inner to outer parts of the endosperm [20]). During seedling growth, the starch in the inner region of the endosperm was completely degraded, but that in the middle and outer regions of the endosperm were partly degraded. The residual starch is located in the peripheral region of the aggregate, elongated, and hollow starch granules [14]. In the present study, though the aggregate, elongated, and hollow starch granules were not detected in the GTR endosperm, the starch residual was also detected in the outer part of the compound starch in the peripheral region of the endosperm (Figure 9(d1–d3)). The above results indicated that starch in different regions of the endosperm or granules had different structures when the SBE expression was inhibited.

### 2.5. Characterization of Endosperm Starch during Seedling Growth

**Figure 10:**
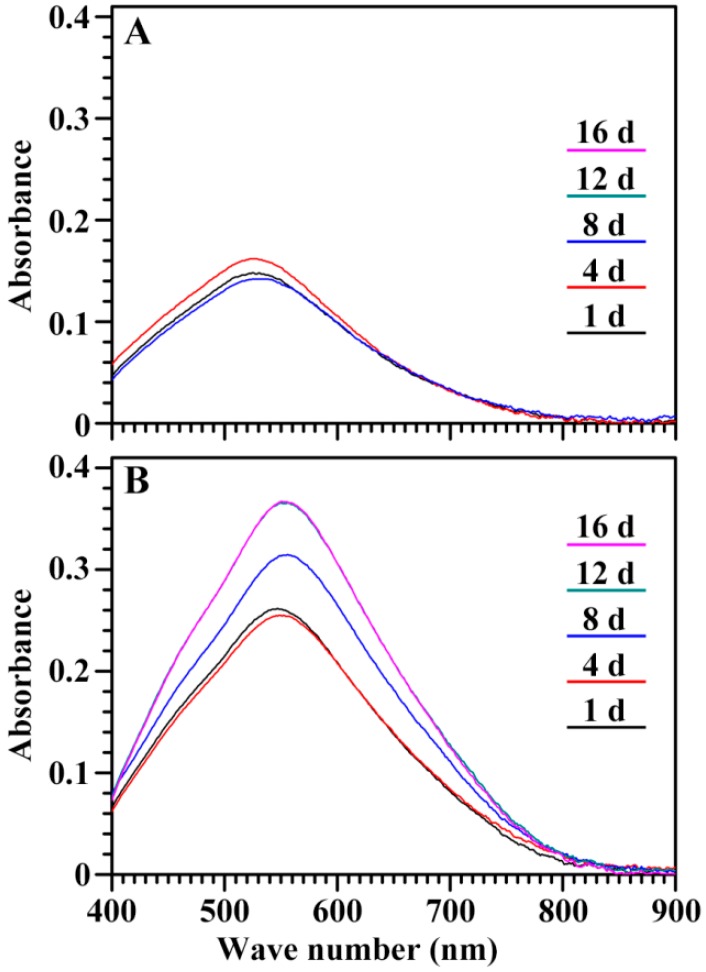
The iodine absorption spectrum of endosperm starch during seedling growth. (**A**) GLXN, (**B**) GTR.

Residual starch in seed was isolated during seedling growth. The iodine absorption spectrum of the starch is shown in Figure 10. During seedling growth, the spectrum had no significant change in the GLXN starch, but exhibited a significant difference in GTR starch. The maximum absorption wavelength (λmax), blue value (BV, absorbance at 680 nm), and the optical density 620 nm/550 nm ratio (OD 620/550) of starch are presented in Table 1. They were similar among the starches from GLXN seeds of different DAIs, but the λmax and OD 620/550 gradually increased and the BV significantly increased from 4 to 12 DAI in the GTR residual starch. Iodine can bind the amylose and amylopectin branch-chains, producing starches having different iodine absorption spectra due to the different contents of amylose and the branch-chain length of amylopectin. The λmax can reflect the chain length of amylose and amylopectin, the OD620/550 can indicate the relative content of the longer branch-chain segments in starch, and the BV can exhibit an iodine affinity with a high value, having a high amylose content or long amylopectin branch-chain [21]. The present study showed that the starches from the GLXN seeds of different DAIs had similar iodine absorption spectra, indicating that the starch in different regions of the seed was homogeneous in the amylopectin structure. However, the starches from the GTR seeds of different DAIs had different iodine absorption spectra, indicating that the starch in different regions of the seed was heterogeneous in the amylopectin structure and that the starches with short branch-chains degraded more rapidly than the those with long branch-chains. In normal barley and rice seedling growth, amylose and amylopectin were simultaneously degraded; however, in high-amylose barley and rice with inhibition of SBE expression, the amylose is preferably degraded compared to the amylopectin, and the long branch-chains of amylopectin were more resistant to hydrolysis than the short branch-chains during seedling growth [10,14].

**Figure 11:**
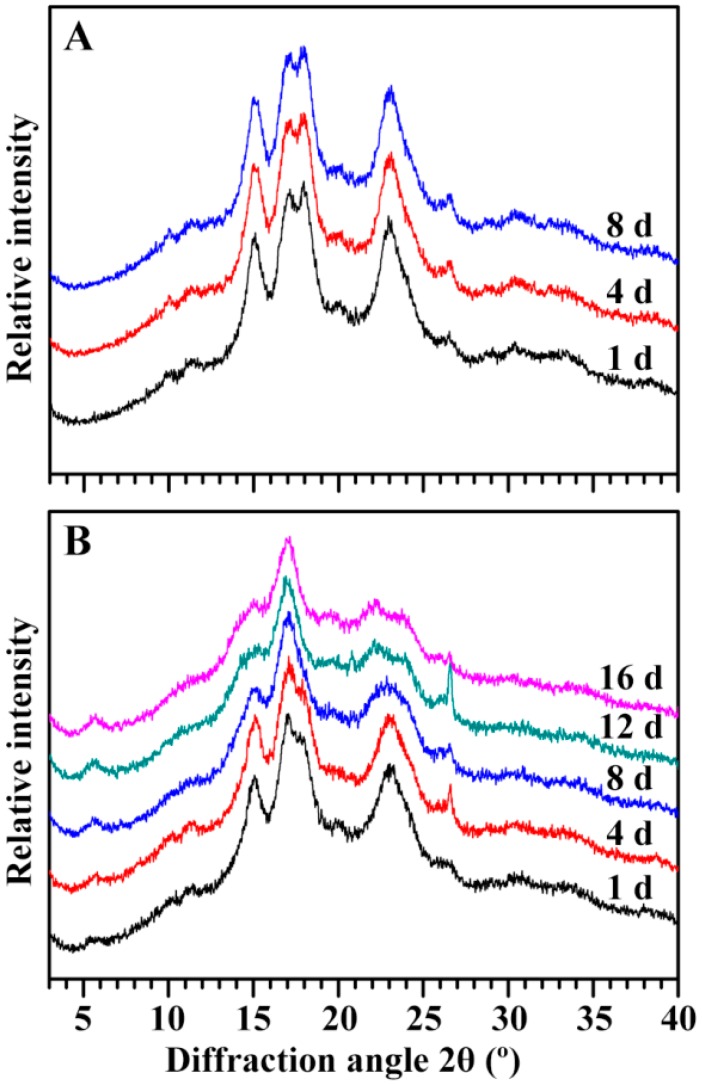
The XRD pattern of endosperm starch during seedling growth. (**A**) GLXN, (**B**) GTR.

The X-ray diffraction (XRD) patterns of starches are shown in Figure 11. Starches are usually classified into the A-, B-, and C-type according to their XRD patterns. The A- and B-type starch contains A- and B-type crystallinity, respectively, and the C-type starch has both the A- and B-type crystallinities [22,23]. No changes were detected in the starches from the GLXN seeds of different DAIs, which exhibited strong diffraction peaks at about 15° and 23° 2θ, and an unresolved doublet at around 17° and 18° 2θ, indicating a typical A-type crystallinity [22]. For GTR, significant changes were found in the starches from the seeds of different DAIs. The starch from the GTR seed of 1 DAI had a weak diffraction peak at 5.6° 2θ, a characteristic peak of the B-type crystallinity, and an obvious shoulder peak at 18° 2θ, indicating that the starch in the GTR seed of 1 DAI had A- and B-type crystallinity and that the A-type crystallinity was higher than the B-type crystallinity, resulting in a C_A_-type starch. With the seedling growth, the shoulder peak at 18° 2θ gradually becomes weak and vanishes, the peak at 23° 2θ gradually widens and becomes two peaks at 22° and 24° 2θ, indicating that the starch changed from the C_A_-type to the B-type with the growth of the seedling. A similar phenomenon has been reported in high-amylose rice with the inhibition of the SBE expression [14]. The short branch-chains and closed branching points of amylopectin can favorably form A-type crystallinity, and the long branch-chains and distant branching points of amylopectin form B-type crystallinity [24]. The long branch-chains of amylopectin increased the resistance to in situ degradation [10,14].

**Figure 12:**
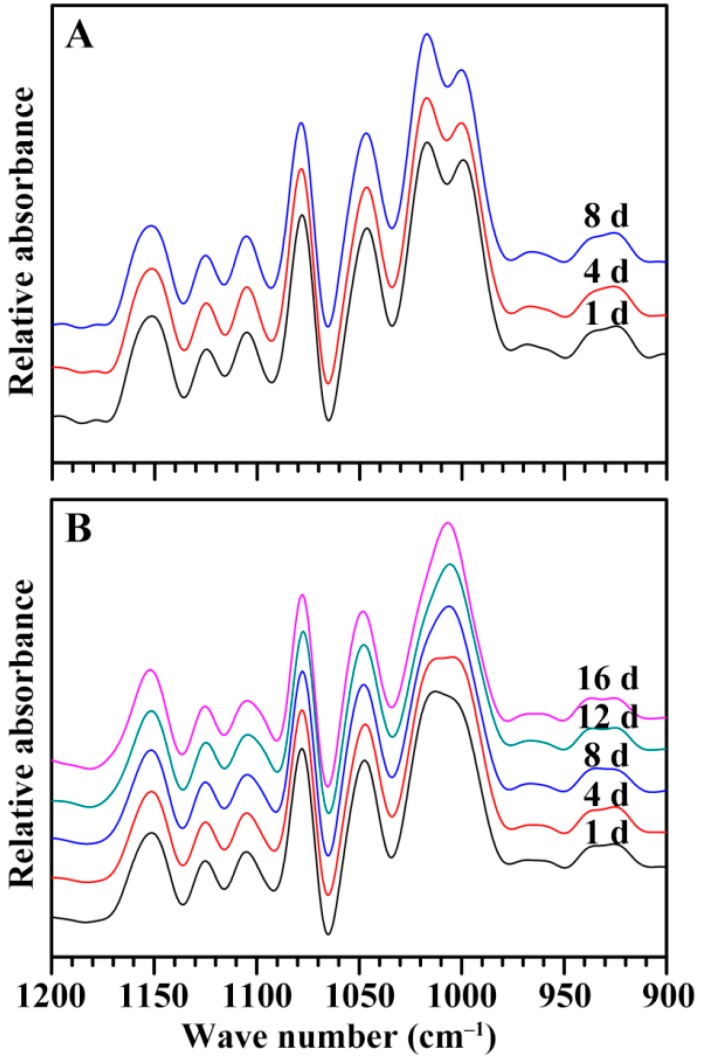
The ATR-FTIR pattern of endosperm starch during seedling growth. (**A**) GLXN, (**B**) GTR.

The attenuated total reflectance-Fourier transforms infrared (ATR-FTIR) spectra of starches are shown in Figure 12. Starch has an amorphous and ordered structure, and the FTIR peaks at 1045 and 1022 cm^−1^ are associated with the ordered and amorphous regions in starch, respectively [25]. For GLXN seeds, the spectra of the starches had no significant change during the seedling growth (Figure 12A), but for GTR seeds, the peak at 1022 cm^−1^ gradually decreased (Figure 12B). The present results indicated that the ordered and amorphous structures in GLXN starch were simultaneously degraded, but the amorphous structure was degraded faster than the ordered structure in the GTR starch. Similar phenomena have also been reported in high-amylose rice with the inhibition of the SBE expression [14].

## 3. Materials and Methods

### 3.1. Plant Materials

The *japonica* waxy rice cultivar GLXN and its derived transgenic rice line GTR were used in this study. The GTR was generated by the antisense RNA inhibition of both SBEI and SBEIIb [9]. They were cultivated in a closed transgenic experimental field in Yangzhou University, Yangzhou, China. Mature grains were used as experimental materials.

### 3.2. Germination of Rice Grain and Seedling Growth

Rice grains were imbibed in deionized H_2_O at 28 °C in the dark for 2 days with changes of water three times a day. The germinated grains were transferred into a 96-well plate with the embryo up and it continued to grow at 28 °C in the dark with the lower two-thirds of the grain immersed in deionized H_2_O. During seedling growth, the water was changed every day. The seedlings were taken out at 1, 4, 8, 12, and 16 DAI, and the grains at 1 DAI were used as the control.

### 3.3. Determination of Shoot Height and the Dry Weight of Shoot, Root, Seed, and Endosperm Starch

The length from the culm base to the tip of the longest leaf of the seedling was measured as the shoot height. The shoot and root were separated from the grain and dried at 110 °C for 3 h and 80 °C for 2 days in an oven. The germinated grains with the shoot and root removed were freeze-dried, and then they were dehulled carefully to obtain the seeds. The dry weights of the shoot, root, and seed were weighed. The seeds were ground extensively into flour and filtered using a 100-mesh sieve. The starch content in the flour was measured using a Total Starch Assay Kit (K-TSTA, Megazyme, Wicklow, Ireland), and then converted to the weight of the endosperm starch.

### 3.4. In Vitro Culture of Mature Embryo

The mature embryo was in vitro cultured exactly following the method by Pan et al. [14]. Briefly, after the washing and sterilizing of the dehulled seeds, the embryos of GLXN and GTR were separated from the seeds and cultured simultaneously in the same tissue culture bottle under a 12-h photoperiod at 28 °C. The shoot height was measured at 6 days after in vitro culture.

### 3.5. Preparation and Observation of Section of Whole Seed

The section of the whole seed was prepared following the method by Zhao et al. [26] with some modifications. Briefly, the seed with the embryo was separated carefully from the grains and immersed immediately in the fixation solution (2.5% glutaraldehyde, 0.1 M Na-phosphate butter, pH 7.2) for 48 h at 4 °C. After fixation, the samples were rinsed with 0.1 M phosphate buffer, dehydrated in gradient ethanol, permeated in gradient LR White resin, embedded in pure LR White resin, and polymerized at 60 °C for 48 h. Sections with a 2 µm thickness were prepared under a Leica Ultrathin Microtome (UC7). The sections were stained with periodic acid-Schiff reagent and observed and photographed with an Olympus BX53 light microscope equipped with a CCD camera.

### 3.6. Isolation of Endosperm Starch

The starch was isolated from the seeds following the method by Pan et al. [14]. Briefly, the seeds without embryos were ground extensively in a mortar and pestle and homogenized with water. The starch water slurry was filtered through 100-, 200-, and 400-mesh sieves, successively, and centrifuged at 5000× *g* for 10 min. The starch precipitate was washed 3 times with deionized H_2_O and 2 times with absolute ethanol, freeze-dried, and filtered through a 100-mesh sieve.

### 3.7. Determination of Iodine Absorption Spectrum of Starch

The starch-iodine absorption spectrum was measured following the method by Lin et al. [21]. Briefly, the starch was dissolved in a urea dimethyl sulfoxide solution and stained with an iodine solution. The sample was scanned from 400 to 900 nm with a spectrophotometer.

### 3.8. Crystalline Structure Analysis of Starch

The starch was analyzed using an X-ray powder diffractometer (D8, Bruker, Karlsruhe, Germany) as previously described [14]. The sample was exposed to an X-ray beam at 40 kV and 40 mA, and scanned from 3 to 40° 2θ with a step size of 0.02°.

### 3.9. Ordered Structure Analysis of Starch

The short-range ordered structure of the starch was analyzed using a Varian 7000 Fourier transform infrared (FTIR) spectrometer exactly following the method by Pan et al. [14].

### 3.10. Statistical Analysis

The data reported in all Figures and Tables were mean values and standard deviations. Analysis of variance (ANOVA) by Tukey’s test was evaluated using the SPSS 16.0 Statistical Software Program (IBM Company, Chicago, IL, USA).

## 4. Conclusions

Compared with GLXN, GTR showed a significantly slow seedling growth. The slow degradation of starch in GTR seed restrained the seedling growth. GLXN starch was homogeneous in endosperm and completely degraded gradually from the proximal to the distal region of the embryo and from the outer to the inner part in the endosperm. However, GTR starch was heterogeneous in the endosperm, and the starch located in the outer layer of compound starch in the peripheral region of the endosperm was not completely degraded. During seedling growth, GLXN starch had the same A-type crystallinity and similar ordered structure, but the crystallinity changed from the C_A_-type to B-type and the ordered structure gradually increased in GTR starch. Therefore, the B-type crystallinity located in the outer part of the compound starch had a high resistance to in situ degradation in GTR endosperm and inhibited seedling growth.

## Figures and Tables

**Table 1:** The iodine absorption parameters of starch isolated from the endosperm during seedling growth ^a^.

Time	GLXN			GTR		
	λmax (nm)	OD 620/550	BV (OD680)	λmax (nm)	OD 620/550	BV (OD680)
1 DAI	525.3 ± 1.8a	0.572 ± 0.014a	0.041 ± 0.002a	548.3 ± 2.5a	0.700 ± 0.015a	0.105 ± 0.003a
4 DAI	526.8 ± 1.8a	0.577 ± 0.012a	0.044 ± 0.001a	549.8 ± 1.1a	0.708 ± 0.008a	0.105 ± 0.001a
8 DAI	528.8 ± 2.5a	0.586 ± 0.018a	0.041 ± 0.003a	553.8 ± 0.4a	0.735 ± 0.001a	0.141 ± 0.002b
12 DAI	527.5 ± 0.7a	0.583 ± 0.024a	0.039 ± 0.003a	555.8 ± 2.5a	0.740 ± 0.019a	0.163 ± 0.005c
16 DAI	–	–	–	555.8 ± 3.2a	0.741 ± 0.019a	0.163 ± 0.006c

^a^ Data are means ± standard deviations (*n* = 3). Values in the same column with different letters are significantly different (*p* < 0.05). Data are not detected.
